# Distribution Characteristic of Soil Organic Carbon Fraction in Different Types of Wetland in Hongze Lake of China

**DOI:** 10.1155/2014/487961

**Published:** 2014-05-22

**Authors:** Yan Lu, Hongwen Xu

**Affiliations:** ^1^School of Urban and Environmental Science, Huaiyin Normal University, Huai'an 223300, China; ^2^Jiangsu Key Laboratory for Eco-Agricultural Biotechnology around Hongze Lake, Huai'an 223300, China

## Abstract

Soil organic carbon fractions included microbial biomass carbon (MBC), dissolved organic carbon (DOC), and labile organic carbon (LOC), which was investigated over a 0–20 cm depth profile in three types of wetland in Hongze Lake of China. Their ecoenvironmental effect and the relationships with soil organic carbon (SOC) were analyzed in present experiment. The results showed that both active and SOC contents were in order reduced by estuarine wetland, flood plain, and out-of-lake wetland. Pearson correlative analysis indicated that MBC and DOC were positively related to SOC. The lowest ratios of MBC and DOC to SOC in the estuarine wetland suggested that the turnover rate of microbial active carbon pool was fairly low in this kind of wetland. Our results showed that estuarine wetland had a strong carbon sink function, which played important role in reducing greenhouse gas emissions; besides, changes of water condition might affect the accumulation and decomposition of organic carbon in the wetland soils.

## 1. Introduction

Wetland played a key role in the global cycles of carbon, which had important function in the global carbon balance and climate change mitigation [1–3]. Carbon cycle process in different types of wetland could provide basis for further understanding of the mechanism of carbon sources and sinks in terrestrial ecosystem [4–6]. Decrease of carbon storage and vegetation biomass in wetland soils might release more carbon into atmosphere, thus causing the increase of atmospheric carbon dioxide concentration [7–10]. Meanwhile, global warming could accelerate decomposition of soil organic matter and release of carbon into atmosphere, which would further strengthen the trend of global warming [11, 12]. Original alteration in soil organic carbon pool of wetland occurred mainly on the part easily to be decomposed and mineralized, that is, activated carbon [13]. Soil activated carbon was referred to the part of the organic carbon with poor stability and higher activity for plants and soil microorganisms [14]. Soil microbial biomass carbon (MBC) and dissolved organic carbon (DOC) were important characteristic indicators for expressing the activity of active soil organic carbon pool [15]. Although active organic carbon was just a very small part of total carbon, its sizes and turnover rates were of great importance to the content and recycling of soil available nutrients, and it was directly related to greenhouse gas emissions [16]. Therefore, exploring the changes of soil active organic carbon in different types of wetland was helpful in achieving deep understanding of the characteristics of wetland soil organic carbon and the response to climate change.

Hongze Lake (33°06′N~33°40′N, 118°10′E~118°55′E) is the fourth largest fresh water lake in China. It is located in the northwest of Jiangsu province, and its water area is 1597 km^2^. The lake and the surrounding area have composed a relatively intact inland wetland as typical lake wetland of China. Differences might exist in soil carbon pool due to various hydrological conditions and vegetation for kinds of wetland. Although researches on soil carbon in wetland have been well documented, limited systematic investigations focus on different types of natural lakes wetland soil carbon and their ecological environment effect. In order to study the distribution characteristics of different types of wetland soil active organic carbon about Hongze Lake Wetland, MBC, soluble organic carbon (SOC), DOC, labile organic carbon (LOC), and their ecological environment effects were studied in the present experiment.

The objective of this research was to study the role which different types of wetland soil active organic carbon played in the biogeochemical cycle and thereby to provide a theoretical basis for a good deal of insight into biogeochemical cycle mechanism of wetland soil carbon.

## 2. Material and Methods

### 2.1. Experimental Design

The experimental site is located at Hongze Lake, China. Three sample plots were selected in south, east, and west bank of Hongze Lake Wetland (representing Estuary wetland, flood plain, and out of lake wetland, separately). The research sample point of three types of wetlands was chosen in the middle of April 2012, and the indicators related to surface vegetation distribution and hydrological conditions were observed and recorded. Soil of 0–20 cm depth was collected into a sterile bag by using the multipoint-mix-sampling method; the soil samples were taken back to the laboratory quickly and divided into two parts. One part was refrigerated under 4°C, which was used to measure soil water content, DOC, and MBC. Another part of the soil sample was just put in the air and taken through a 0.25 mm sieve after being dried and grinded. The latter part was applied to the analysis of SOC and LOC.

### 2.2. Measurements

Soil organic carbon was determined by taking potassium dichromate volumetric method. MBC was measured using chloroform fumigation-K_2_SO_4_ extraction method. TOC instrument was used to determine DOC concentration. Content of LOC estimation was performed according to the method of potassium permanganate oxidation.

### 2.3. Data Analysis

The data was analyzed by one-way analysis of variance (ANOVA) followed by Duncan's test at 0.05 significance level to compare the means using SPSS 16.0 for Windows.

## 3. Results

### 3.1. Distribution Characteristics of Soluble Organic Carbon

**Figure 1:**
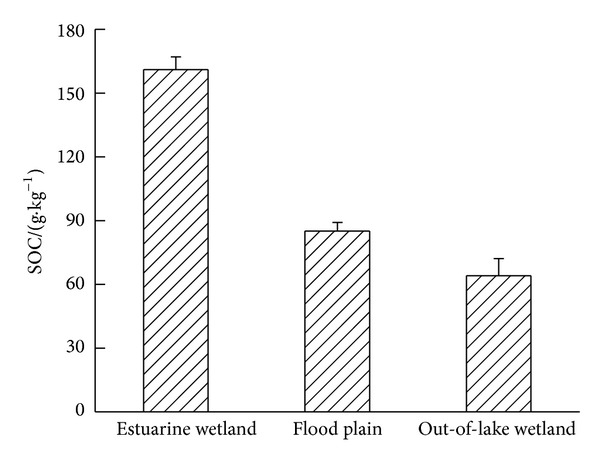
Changes of SOC content under different types of wetland in Hongze Lake of China.

Soil was considered as a carbon source and sink; if organic matter content in soil reduced by one percent, atmospheric CO_2_ concentration would increase by 5 mg·m^−3^. The difference between soil texture and number of vegetation types would lead to variety of input and output of organic matter and causing differences in SOC content.  Figure 1 showed the change trend of SOC content at 0–20 cm depth in three kinds of typical natural wetlands. It also could be seen that the SOC content in the estuary wetland had the peak value with 161.67 g·kg^−1^, which was significantly higher than that of two other types of wetlands. SOC content of wetland soil depended largely on the circulation and decomposition rate of vegetation annually. Estuary wetland was under flooded condition all the year round, resulting in the slow decomposition of organic residue, which caused accumulating of SOC. A seasonal depletion that occurred in flood plain, aerobic microorganisms, and soil enzyme hydrolysis could bring about higher decomposition of organic matter than estuarine wetland when it was not flooding [17]. However, ventilation condition in flood plain was relatively poor compared with out-of-lake wetland. Therefore, the organic carbon content of flood plain was fairly higher than the latter.

### 3.2. Distribution Characteristics of Microbial Biomass Carbon

**Figure 2:**
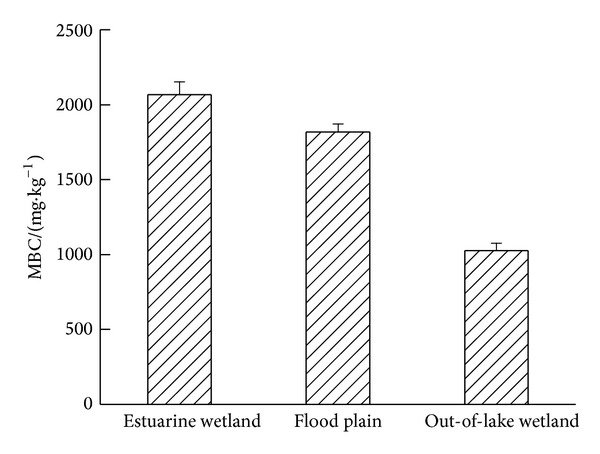
Changes of MBC content under different types of wetland in Hongze Lake of China.

MBC was referred to the internal carbon in live bacteria, fungi, algae, and soil animals whose volume was less than 5–105 *μ*m^3^; it accounted for only a small portion of total carbon in soil; however, it was the most active section in soil organic matter [18]. Besides, it was taken on as the main driving force for decomposition of nutrient pool [19–21]. As an important source of soil nutrients, it correlated closely with cycling of the nutrients such as C, N, P, and S [22]. Changes of MBC were in accordance with SOC, and significant difference of MBC content in soil of three different types of wetland was found in present experiment (Figure 2) (*P* < 0.01). Positive relationship was found between soil MBC content and SOC content of three kinds of wetland (*r* = 0.823, *P* < 0.05). The ratio of MBC and SOC reflected the conversion efficiency from the organic matter into MBC, which could imply the turnover rate of biologically active soil organic carbon pool. The specific value of MBC/SOC of estuary wetland, flood plain, and out-of-lake wetland was 1.28%, 2.12%, and 1.6%, which suggested that flooded condition could inhibit aerobic microbial activity.

### 3.3. Distribution Characteristics of Dissolved Organic Carbon

**Figure 3:**
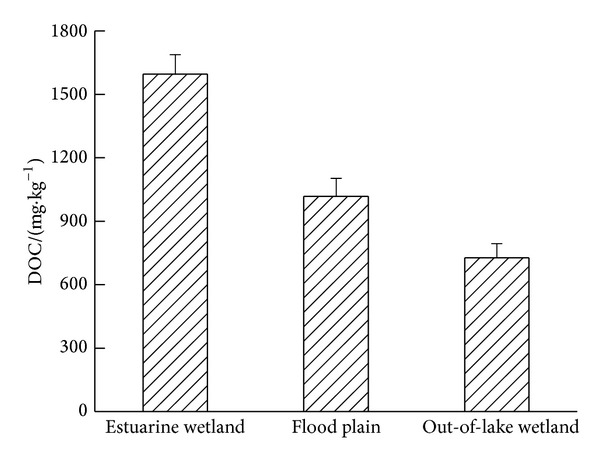
Changes of DOC content under different types of wetland in Hongze Lake of China.

DOC mainly originated from the recent humus of plant litter and soil organic matter, which included a series of organic matter from simple organic acids to the complex of macromolecular substances, such as humic acid and fulvic acid [23–25]. And it was taken as organic carbon source directed using for soil microbes, and affecting the transformation, migration, and degradation of soil organics and inorganics [26–28]. The formation, migration, and transformation of DOC in the wetland had important influence on soil carbon flux [29]. Similar changing trend in DOC and MBC in 0–20 cm of soil appeared in Figure 3, and ANOVA analysis showed that the DOC content of estuarine wetland was significantly higher than that of flood plain and out-of-lake wetland (*P* < 0.01). This was due to the flooded condition of estuary wetland, which could improve the dissolution of soil organic carbon and the dispersion of soil aggregate [30, 31]. Pearson correlation suggested that it was significantly and positively related to DOC and SOC of the three different types of wetland soil (*r* = 0.872, *P* < 0.01), which indicated that SOC was the main source of DOC. The ratio of DOC to SOC of estuary wetland, flood plain, and out-of-lake wetland was 0.09%, 1.19%, and 1.12% individually. The lowest proportion that occurred in estuarine wetland would declare the lower turnover rate of biological active soil organic carbon pool in estuary wetland.

### 3.4. Distribution Characteristics of Labile Organic Carbon

**Figure 4:**
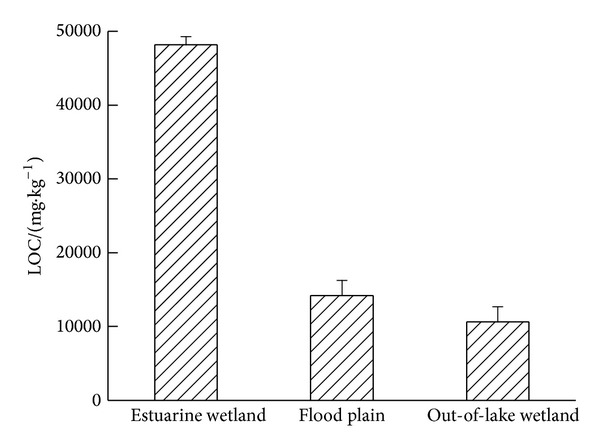
Changes of LOC content under different types of wetland in Hongze Lake of China.

Soil LOC content and the ratio of LOC to SOC were the indexes to reflect the stability of soil carbon. And the two indexes of estuarine wetland soil were significantly higher than that of flood plain and out-of-lake wetland (Figure 4). Positively significant correlation between LOC and SOC in different types of wetland soil was found in present experiment (*r* = 0.952, *P* < 0.01); thus, the quantity of SOC could determine the abundance of LOC. The higher the proportion of LOC/SOC was, the worse the stability was. Special hydrological conditions of wetland would make the anaerobic environment; when hydrological conditions changed from anaerobic into aerobic environment, huge LOC would be decomposed and consumed by microbes [32, 33].

## 4. Conclusions

From this study, we concluded that estuarine wetland had fairly important environmental function in the greenhouse gas emissions reduction and the climate change mitigation. And we supported the speculation that variation in water condition would have a real impact on the accumulation and decomposition of organic carbon in the wetland soils.
